# The association between betel quid use and oral potentially malignant and malignant disorders in Southeast Asian and Pacific regions: a systematic review and meta-analysis with GRADE evidence profile

**DOI:** 10.3389/froh.2024.1397179

**Published:** 2024-05-23

**Authors:** Aula Jasim, Xia Li, Alfini Octavia, Indrayadi Gunardi, Leonard Crocombe, Elizabeth Fitriana Sari

**Affiliations:** ^1^ Dentistry and Oral Health Discipline, Department of Rural Clinical Science, La Trobe Rural Health School, Bendigo, VIC, Australia; ^2^ Department of Mathematical and Physical Sciences, La Trobe University, Bundoora, VIC, Australia; ^3^ Faculty of Dentistry, Universitas Muhammadiyah Yogyakarta, Yogyakarta, Indonesia; ^4^ Department of Oral Medicine, Faculty of Dentistry, Universitas Trisakti, West Jakarta, Indonesia; ^5^ Medicine and Health Science, University of Papua New Guinea, Port Moresby, Papua New Guinea

**Keywords:** betel quid, stem inflorescence, areca nut, oral submucous fibrosis, oral cancer

## Abstract

**Background:**

Betel quid (BQ) chewing is a prevalent habit in the Asian and Pacific regions. It is deeply intertwined within cultural customs, and has been reported to result in oral potentially malignant disorders (OPMDs) and malignant disorders (MDs).

**Objective:**

We aim to present a summative and broad overview of the burden that BQ chewing has imposed on the residents of the Southeast Asian, Pacific, and Australasian regions, allowing us to quantify the level of impact it is currently causing on the risk of people developing oral cancer.

**Methods:**

This scoping review and meta-analysis screened databases such as PubMed, MEDLINE, and Google Scholar for publications that investigated the association between BQ and OPMDs and MDs. The search strategy involved MeSH headings relating to BQ, OPMDs, and MDs, and a search for results during the period between January 2010 and June 2023 within the set geographical boundaries of the Southeast Asian and Pacific regions. This systematic review was reported in accordance with the Preferred Reporting Items for Systematic Reviews and Meta-Analyses Statement for Scoping Reviews (PRISMA-ScR). R software was used to screen outliers. The included studies were further analysed using the Grading of Recommendations Assessment, Development and Evaluation (GRADE) system.

**Results:**

Nine articles (*n* = 19,312 participants) presented odds ratio outcomes from 11 regionally different study groups. We indicated a strong correlation between BQ chewing and the increased risk of OMPDs and MDs. The risk was quantified through meta-analyses with an odds ratio (OR) of 8.18 (5.27–12.72) and an increased OR of 9.93 (7.36–13.39) when the outlier was removed. BQ chewing was further identified within various Australian communities and discovered to be produced locally in North Queensland.

**Discussion:**

A meta-analysis of two outcomes revealed substantial heterogeneity and minor evidence of publication bias, thus the association effect was included with and without these articles. The overall GRADE quality of evidence ranged from moderate to very high and highlighted five studies with a high level of imprecision.

**Conclusion:**

The lingering high prevalence of BQ in the Southeast Asia and Pacific regions, as well as its rising acceptance among non-ethnic Australians, is alarming and requires prompt and rigorous intervention to prevent the risk of oral cancer.

**Systematic Review Registration:**

PROSPERO (CRD42023429694).

## Introduction

Betel quid (BQ) chewing is an ancient practice originating from cultures across Africa, Asia, and the Pacific, and BQ is chewed by approximately several hundred million adults and children (1). The practice involves chewing or placing a BQ mixture in the mouth, where it remains in contact with the oral mucosa for long periods of time. While BQ constituents and preparations vary across geographic regions, the two ingredients that are consistently used are areca nut, a seed from the *Areca catechu* palm, and a leaf or flower (inflorescence) of the *Piper betel* plant. Areca nut may be consumed in a range of preparation types such as unripe, ripe, roasted, processed, or fermented, and additives such as slaked lime, spices, and tobacco may be included (2, 3). The custom is interwoven with social and religious practices, carrying cultural significance in the regions of Southeast Asia (SEA) and the Pacific Islands. However, it is now becoming widespread across other countries due to migration and cultural sharing (3–5).

Markedly, BQ has become associated with several mucosal diseases, including oral potentially malignant disorders (OPMDs), most commonly, oral submucous fibrosis (OSF), leukoplakia, erythroplakia, and oral lichen planus (OLP). These are pre-cancerous conditions with an elevated risk of transforming into head and neck cancers (3, 6).

OSF, the OPMD most commonly associated with BQ, is a collagen-related metabolic disorder that initiates in the juxta-epithelial tissues as fibrosis, distributing in the oral cavity over time. It presents as blanching, depapillation of the tongue, ulceration, and taste intolerance. As it progresses, OSF causes a rigidity of oral structures, resulting in trismus and dysphagia. Having a high malignancy transformative rate, OSF-affected tissue may exhibit dysplastic changes over time (6). The most common oral MD is oral squamous cell carcinoma (OSCC), which infamously has a low 5-year survival rate of approximately 50% (5, 6).

The aim of the present study is to evaluate existing literature regarding BQ use and its association with OPMDs and/or MDs. We hope that our findings will present a summative and broad overview of the burden that BQ chewing has imposed on the residents of the SEA, Pacific, and Australasian regions, allowing us to quantify the level of impact it is currently having on the risk of people developing oral cancer. We also hope that our review will underline the detrimental impact of BQ chewing behaviour. We anticipate that it will throw light on the covert creep of the habit to neighbouring countries, where the impact of BQ may have been previously underestimated. Ultimately, we expect that the results of this systematic review will allow us to make recommendations for future research and intervention on the prevention of oral cancer.

## Methods

### Search protocol and study selection

This systematic review and meta-analysis are reported in accordance with the Preferred Reporting Items for Systematic Reviews and Meta-Analyses Statement for Scoping Reviews (PRISMA-ScR). The review protocol was submitted to PROSPERO (Ref: CRD42023429694). We searched PubMed and MEDLINE by considering them primary databases and supplemented the search by including eligible grey literature from Google Scholar during the period between 1 January 2010 and 15 June 2023. The search string that was used was “[Areca OR (betel quid)] AND (Neoplasms OR Pre-cancerous Condition) AND [Australasia OR (Southeastern Asia) OR (Pacific Islands)]”, with appropriate “MeSH” and subject headings relevant to the database being employed. It was decided to search “betel quid” as a keyword as it was not encompassed in the (Areca) entry terms/references (Supplementary Table S1). As such, results were limited to the geographic region inclusion criterion before the first hit. The search was further limited to studies in English where full text was accessible, or the abstract contained sufficient information to be included in the meta-analysis and review. The review involved only human subjects. The included studies were also assessed using the Grading of Recommendations Assessment, Development and Evaluation (GRADE) system and by screening outliers applying R software functions. An expert in the field of oral medicine and BQ-related diseases was consulted about the search strategy prior to the commencement of the screening process.

### Eligibility criteria

We included studies that were well designed and studied the association between BQ use with or without tobacco and OPMDs and/or MDs. Studies were required to be made from data collected from the SEA, Australasian, and Pacific Island countries listed in Supplementary Table S1. As there was a variety of interpretations regarding which countries represent SEA, the construct of the United Nations was adopted. The excluded studies were: existing systematic reviews and meta-analyses; studies pertaining to the association of BQ with factors other than OPMDs and MDs; studies that investigated the association between OPMDs and MDs and oral habits such as alcohol and smoking, without adequately considering BQ exposure; and studies with a general interest in BQ, although not showing a clear association between BQ and the pathogenesis of OPMDs and MDs. For the meta-analysis, we further excluded studies that did not involve an odds ratio (OR) exclusively reporting the association of risk between BQ and OPMDs/MDs.

### Study selection and data extraction

Titles and abstracts from the results were screened against the inclusion and exclusion criteria, filtering out those that were clearly irrelevant. After this phase of screening, the full texts of the remaining articles were obtained, and, in collaboration with the oral medicine specialist (EF), they were thoroughly analysed and matched against the selection criteria through Covidence. Covidence was used to import search results for independent screening by two reviewers (AJ and ES). According to the website restrictions, all decisions were made by using the anonymous votes of the two researchers until a unanimous decision was taken. In the event of disagreements on decisions, a discussion was undertaken until a consensus was reached.

Google Scholar was additionally searched for grey literature as a supplementary database to find articles not included in the PubMed or MEDLINE library databases. Also being aware of its limitations and a lack of a filtering option to yield specific search results, we applied the same time limit for the search, and only the first 50 results were screened until there was no new and/or relevant information to be obtained. The search string was “betel quid areca nut cancer,” followed by each country or region of interest. This string was used because “betel quid” and “areca nut” are sometimes non-interchangeable terms in the literature and also because we were seeking articles that discussed BQ purely from the carcinogenesis standpoint.

Full texts were scanned against selection criteria and included as appropriate. The reference lists of all the included studies were manually searched for additional relevant studies until no further relevant publications were found. Finally, parameters from each study were obtained, including the name of the first author, year of publication, region where the study was conducted, BQ ingredients when available, study size, the Crude OR (CrOR), and the Adjusted OR (AdjOR) associating BQ with OPMD/MD risk.

The meta-analysis data selection excluded studies that lacked a defined, adjusted OR. In addition, in cases where studies explored BQ in a multitude of combinations with smoking or chewing tobacco and drinking alcohol, “chewing BQ only” was the selected outcome. In studies that compared “BQ abusers” against “occasional chewers,” the former was included in the meta-analysis.

### Data synthesis and meta-analysis

From the nine studies included in the final meta-analysis, the following data were extracted where available: country of study, investigator name and year of publication, study design, study size, and reported adjusted OR with 95% confidence interval (CI) and *p*-value. The quality of the studies was graded according to guidelines stipulated by GRADE (7, 8). We utilised R software (version 4.3.1) with meta-analysis packages *meta* and *dmetar* to perform the meta-analysis and combine the adjusted OR values (9). We tested for heterogeneity among studies through the *I*^2^ test, which yielded a moderate to high heterogeneity and required the use of a random effects model as part of the produced forest plot. Outliers were identified and sensitivity testing was performed by omitting studies to observe the significance of the changes in results. Finally, a funnel plot was produced, and Egger's test was used to test for the presence of publication bias.

## Results

### Overview of the search process

**Figure 1:**
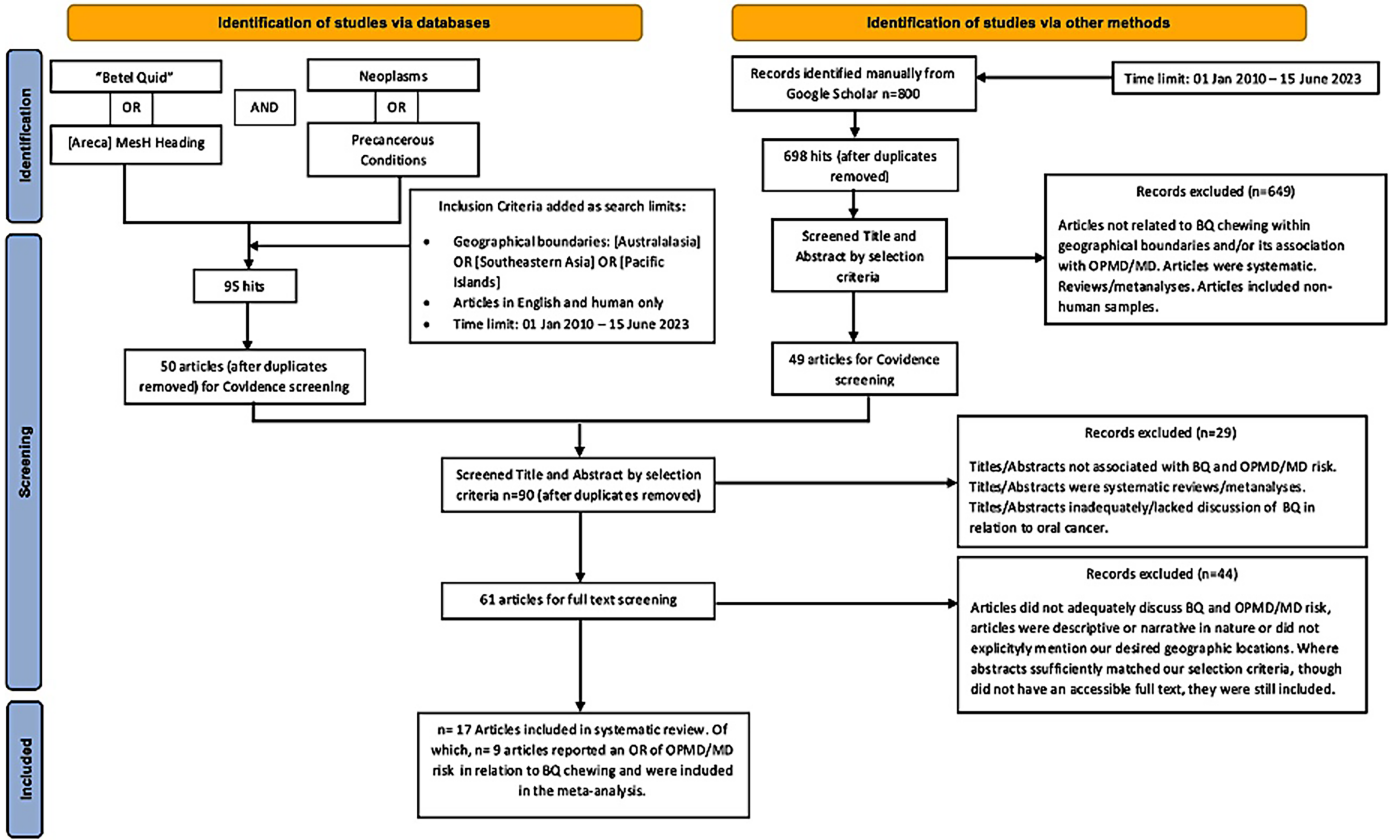
A flow chart of the selection process according to PRISMA 2020.

The search process was a combination of two separate search types. The first was a database search, which retrieved 95 studies after the inclusion criteria were combined within the search strategy, out of which 45 duplicates were removed. The remaining 50 articles were imported into Covidence. The second search type identified 800 records from Google Scholar, out of which 102 were duplicates and 649 did not meet the selection criteria, while 49 studies were imported into Covidence. After duplicates were automatically removed, screening by title and abstract, and then by full text where relevant, was performed by using Covidence's voting system. Ultimately, 17 articles were included in the systematic review, out of which nine met the eligibility criteria of the meta-analysis (Figure 1 and Table 1). The quality of the observational studies included in the meta-analysis was also graded according to the GRADE guidelines (7, 8, 17). The overall GRADE quality of evidence from screened studies ranked from moderate to very high. Out of 11 studies, three had a high level of imprecision due to wide confidence intervals, and in two, the imprecision was attributed to their very small sample sizes, as can be seen in Table 2.

**Table 1:** Results.

Study country	Investigator study type	Constituents (W/WO tobacco)	Study size	Crude OR, 95% CI, *p*-value	AdjOR, 95% CI, *p*-value	Adjustment for factors
Taiwan	Lin et al. (10) Prospective cohort study	Betel quid only	10,487	None reported	11.95 (3.54, 40.33), *p* < 0.001	Multivariate logistic regression model
Taiwan	Lee et al. (3) Cross-sectional study	Areca nut + betel leaf (84.4%) Areca nut + inflorescence (8%) Areca nut + stem (3.3%)	736	None reported	41.8 (7.8–222.4) *p* < 0.005	Mean: regression coefficients OR: multiple linear and logistic regression modelling with backward elimination and forward selection procedures Adjusted for age, tobacco smoking and alcohol drinking. Confounders: Age
Mainland China		Dried areca nut husk marinated in flavoured additives and lime. No tobacco	1,225	None reported	36.6 (9.3–143.8) *p* < 0.005	
Indonesia		BQ without tobacco (29.7%) BQ + tobacco (70.4%)	965	None reported	14.4 (6.3–32.9) *p* < 0.005	
Thailand	Loyha et al. (11) Retrospective study	BQ mixture including lime	104	4.11 (1.88–8.93) *p* < 0.001	9.01 (3.83–21.22) *p* < 0.001	1) Logistics regression involving a univariate analysis of risk factors 2) Multivariable analysis for factors occurring at the same time 3) Logistic regression a model with backward selection
Indonesia	Amtha et al. (12) Retrospective study	Betel leaf + Areca nut + lime + tobacco	123	4.19 (1.05–16.82) *p* = 0.043	4.59 (1.11–18.91) *p* = 0.035	Univariate logistical regression to obtain the crude ratio and then multivariate logistic regression for values where *p* > 0.250 in the univariate model. Adjusted for alcohol, smoking, and dietary pattern
Myanmar	Zaw et al. (2) Cross-sectional study	BQ without tobacco	542	6 (2–17) *p*-values not available	5.7 (1.4–22.9) *p*-values not available	Adjusted for age, sex, betel chewing, and alcohol drinking from a multiple logistic regression model
Thailand	Juntanong et al. (13) Cross-sectional study	Not described	2,300	4.28 (1.84–11.55) *p* < 0.001	8.81 (3.17–24.45) *p* < 0.001	Conditional logistic regression. Univariate analysis. Multivariate analysis with backward elimination where factors *p* < 0.25 had statistical significance in previous studies
Indonesia	Sari and Cirillo (14) Cross-sectional study	Not described	973	None reported	8.16 (5.25–12.68)	Not described
Thailand	Worakhajit et al. (15) Case–control study	Areca nut + betel leaf + Tobacco + flavours (turmeric)	1,448	Current: 6.91 (5.43–8.79) *p* < 0.001 Former: 6.89 (3.37, 14.10) *p* < 0.001	4.65 (3.29–6.58) *p* < 0.001 (current/former)	Logistic regression analysis. Univariate analysis and all *p* < 0.2 selected for multivariate analysis
Thailand	Klongnoi et al. (16) Case–control study	Not described	409	3.191 (2.083–4.887) *p* < 0.001	2.925 (1.753–4.880) *p* < 0.001	Multiple logistic regression

W, with; WO, without; OR, odds ratio; AdjOR, adjusted odds ratio.

**Table 2:** GRADE evidence profile: the association between BQ chewing and OPMDs/OMs.

Quality assessment						Summary of findings			
Studies	Limitations	Inconsistency	Indirectness	Imprecision	Publication bias	Study size	Odds ratio (95% CI)	Quality of the evidence (GRADE)	Comment
Lin et al. (10)	No serious limitations	Consistent	Direct	Serious (−1)	Undetected	10,487	11.95 (3.54–40.33)	⊕ ⊕ ⊕, Moderate	Imprecision due to a wide CI (−1), large effect size (+1), and positive gradient (+1)
Lee et al. (3) Taiwan	No serious limitations	Consistent	Direct	Serious (−1)	Undetected	736	41.8 (7.8–222.4)	⊕ ⊕ ⊕ ⊕, High	Imprecision due to a wide CI (−1), large effect size (+1), and positive gradient (+1), adjusted for confounding variables (+1)
Lee et al. (3) China	No serious limitations	Consistent	Direct	Serious (−1)	Undetected	1,225	36.6 (9.3–143.8)	⊕ ⊕ ⊕ ⊕, High	Imprecision due to a wide CI (−1), large effect size (+1), and positive gradient (+1), adjusted for confounding variables (+1)
Lee et al. (3) Indonesia	No serious limitations	Consistent	Direct	No high imprecision	Undetected	965	14.4 (6.3–32.9)	⊕ ⊕ ⊕ ⊕, High	Large effect size (+1) and positive gradient (+1), adjusted for confounding variables (+1). Decision to grade as high as to match other studies in the same article
Loyha et al. (11)	No serious limitations	Consistent	Direct	Serious (−1)	Undetected	104	9.01 (3.83–21.22)	⊕ ⊕ ⊕, Moderate	Small sample size (−1) and positive gradient (+1), adjusted for confounding variables (+1)
Amtha et al. (12)	No serious limitations	Consistent	Direct	Serious (−1)	Undetected	123	4.59 (1.11–18.91)	⊕ ⊕ ⊕, Moderate	Small sample size (−1) and positive gradient (+1), adjusted for confounding variables (+1)
Zaw et al. (2)	No serious limitations	Consistent	Direct	No high imprecision	Undetected	542	5.7 (1.4–22.9)	⊕ ⊕ ⊕ ⊕ ⊕, Very high	Large effect size (+1) and positive gradient (+1), adjusted for confounding variables (+1)
Juntanong et al. (13)	No serious limitations	Consistent	Direct	No high imprecision	Undetected	2,300	8.81 (3.17–24.45)	⊕ ⊕ ⊕, Moderate	Large effect size (+1) and positive gradient (+1), adjusted for confounding variables not stated clearly (−1)
Sari and Cirillo (14)	No serious limitations	Consistent	Direct	No high imprecision	Undetected	973	8.16 (5.25–12.68)	⊕ ⊕ ⊕ ⊕ ⊕, Very high	Large effect size (+1) and positive gradient (+1), adjusted for confounding variables (+1)
Worakhajit et al. (15)	No serious limitations	Consistent (heterogeneity explained by population variance)	Direct	No high imprecision	Unlikely (Egger's test negative to asymmetry, although the funnel plot appears asymmetrical)	1,448	4.65 (3.29–6.58)	⊕ ⊕ ⊕ ⊕ ⊕, Very high	Large effect size (+1) and positive gradient (+1), adjusted for confounding variables (+1)
Klongnoi et al. (16)	No serious limitations	Consistent (heterogeneity explained by population variance)	Direct	No high imprecision	Unlikely (Egger's test negative to asymmetry, although the funnel plot appears asymmetrical)	409	2.925 (1.753–4.88)	⊕ ⊕ ⊕, Moderate	Large effect size (+1) and positive gradient (+1), adjusted for confounding variables not stated clearly (−1)

All journals assessed in this article are observational studies and initially ranked as “low”, further upgraded, or downgraded for quality of evidence according to the GRADE system.

### The relation between BQ chewing and OPMD/MDs

**Figure 2:**
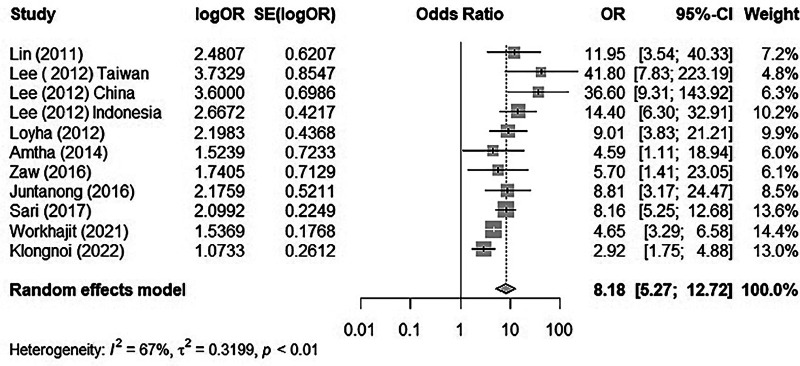
A forest plot of BQ chewing and its association with OPMD/MDs.

There was substantial heterogeneity between each study (*I*^2^ = 67%, tau^2 ^= 0.3199, *p* < 0.01). Thus, we used the random effect model to merge the adjusted ORs and form a forest plot. The meta-analysis results suggested that chewing BQ placed the chewer at a 7.18-fold risk of oral cancer (OR = 8.18, 95% CI: 5.27–12.72; Figure 2).

### Outliers and influential factors

We used the find.outliers function of R software to detect one outlier, “Klongnoi (2022).” The function automatically reran the analysis, while excluding the identified study. Based on the output, we found that the *I^2^ *= 55.8 heterogeneity shrank considerably when this study was excluded. The combined OR increased (OR = 9.14, 95% CI: 6.13–13.61).

Baujat plots are diagnostic plots produced to detect studies that greatly contribute to the heterogeneity of a meta-analysis on the x-axis and its influence on the pooled effect size along the y-axis. Studies on the right side of a graph are potentially relevant due to the factor of high heterogeneity, while studies on the upper right corner are considered influential due to their large impact on estimated heterogeneity and pooled effect. In our study, the identified one is “Klongnoi (2022)” (Supplementary Figure S1).

We see an interesting pattern in our data: while most values are concentrated in a cluster with relatively medium effects and high heterogeneity, the distribution of *I*^2^ values is heavily right-skewed and bimodal (Supplementary Figure S2). There seems to be some study combinations for which the estimated heterogeneity is much lower, but where the pooled effect size is similar. Having seen the effect size heterogeneity pattern in our data, the most important question that arises is: which studies cause this shape? To identify them, three tests were carried out: K-means (Supplementary Figure S3), DBSCAN (Supplementary Figure S4), and a Gaussian Mixture Model (Supplementary Figure S5). Both K-means and Gaussian Mixture Model identified studies “Worakhajit 2021” and “Klongnoi (2022)” as potential outliers, while DBSCAN identified “Sari (2017)” as a potential outlier.

**Figure 3:**
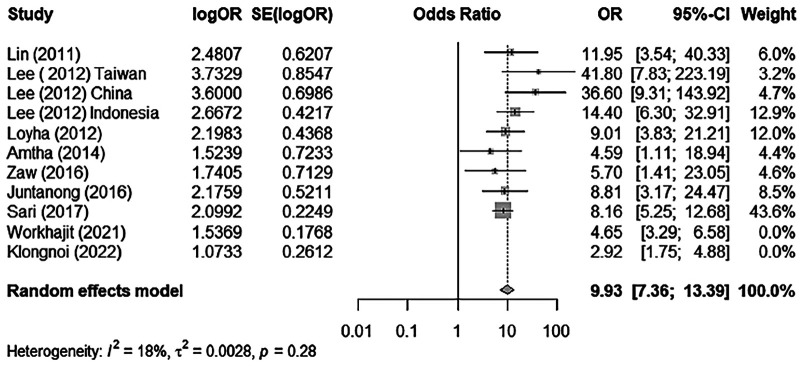
A forest plot of BQ chewing and its association with OPMD/MDs excluding outliers/influential cases.

Thus, if we select “Worakhajit 2021” and “Klongnoi (2022)” as potential outliers and run the meta-analysis without these studies, we obtain the following results (Figure 3 and Table 3).

**Table 3:** Summary of meta-analysis.

Analysis	OR	95% CI	*p*-value	*I* ^2^	95% CI
With 11 papers	8.18	5.27–12.72	<0.0001	67%	37.9%–82.5%
Infl. Out. cases removed (9 papers)	9.93	7.36–13.39	<0.0001	18%	0.0%–60.1%

### Sensitivity test

We tested for the sensitivity of results by omitting a single study each time to identify its influence on overall ORs and heterogeneity. These results were not significantly altered when any part of the study was omitted, indicating that there was little impact from individual studies on the overall positive association between BQ and OPMDs/MDs (Supplementary Figures S6, S7).

### Assessment of risk of publication bias

**Figure 4:**
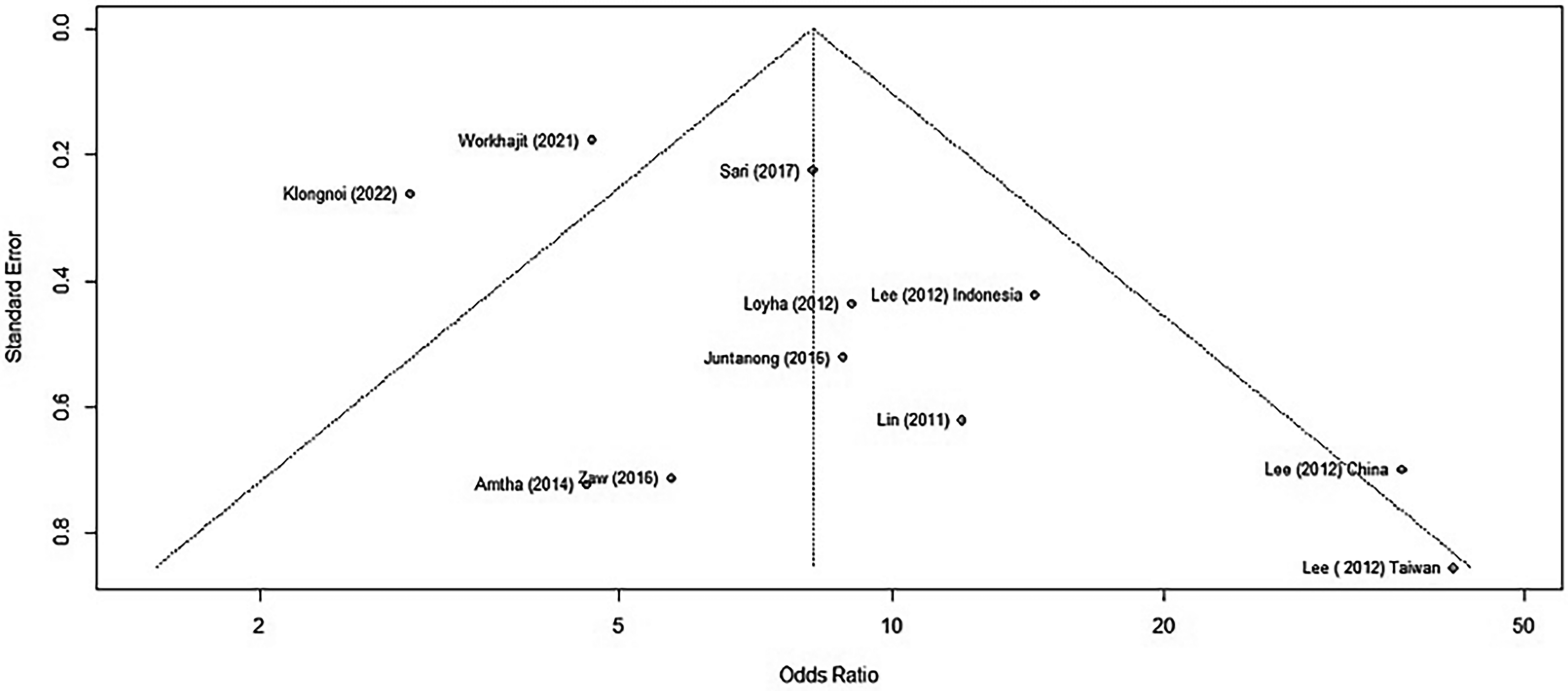
A funnel plot of studies reporting BQ chewing and its association with OPMD/MDs.

We assessed for publication bias by generating a funnel plot on R software. Previously, the funnel plot generated without removing the two outlier studies produced an asymmetrical plot, clearly identifying the outlier studies (Figure 4).

**Figure 5:**
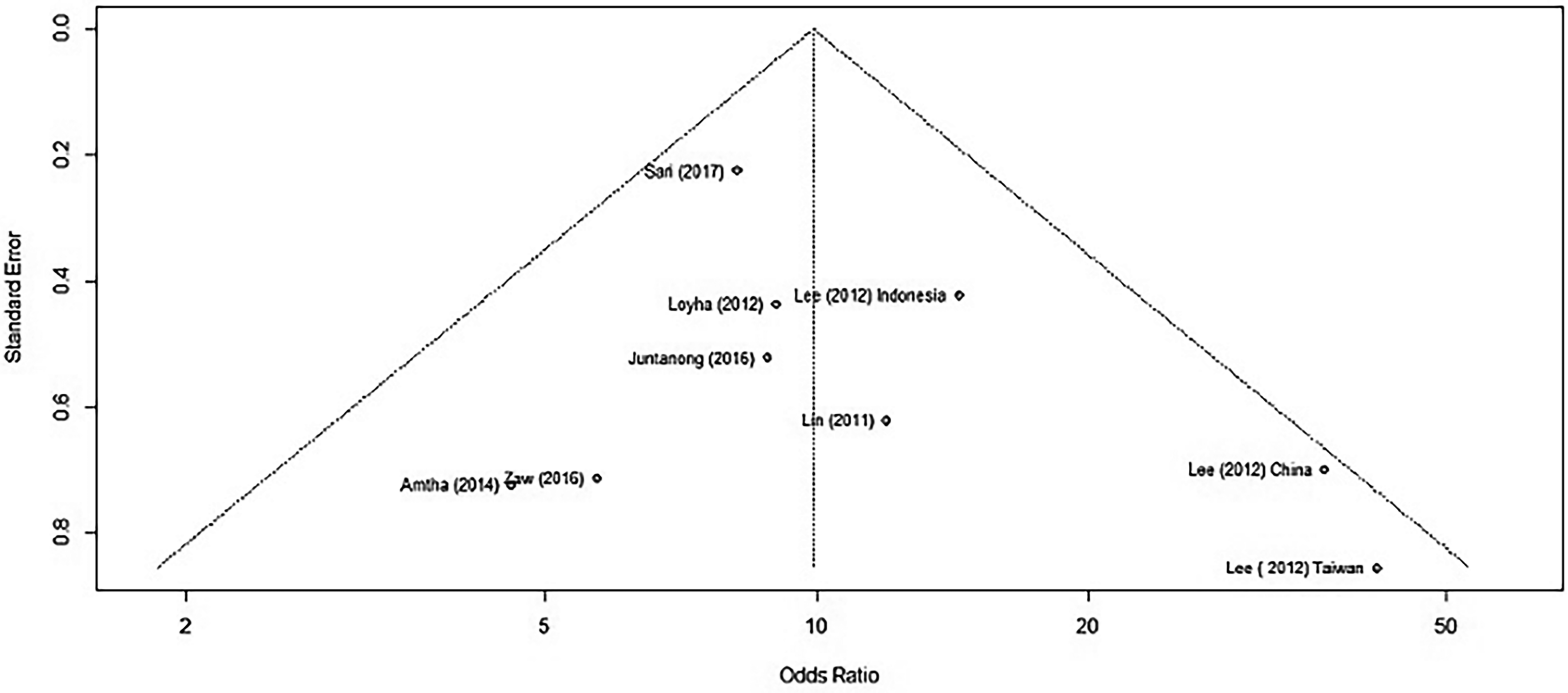
A funnel plot of studies reporting BQ chewing and its association with OPMD/MDs excluding outliers/influential cases.

When using only the nine published results, we generated a new funnel plot (Figure 5).

We applied Egger's test on the funnel plot, which is a linear regression of the effects on their standard errors weighted by their inverse variance. The test of intercept did not detect any signs of asymmetry, which may indicate that there is no publication bias within those studies (*p* = 0.2777) (Supplementary Figure S8).

### Meta-analysis reporting

The meta-analysis run by R software revealed a positive relationship between BQ chewing and OPMDs and/or oral cancer. These two influential factors stood out as potential outliers in the studies, labelled as “Worakhajit 2021” and “Klongnoi (2022).” When omitting these studies, the rate of heterogeneity decreased drastically from a 67% moderate to high heterogeneity to a low heterogeneity of 18%. The pooled OR estimate of the original meta-analysis was 8.18 (95% CI: 5.27–12.72), which increased to 9.93 (95% CI: 7.36–13.39) when omitting the two potential outliers/influential factors. This, as well as sensitivity testing, all confirmed an unquestionable positive association between BQ chewing and OPMDs/MDs.

Generally, heterogeneity is expected in a meta-analysis such as this. As studies do not have identical empirical settings, as they are conducted in different countries and using varying BQ mixtures and chewing methods, and with added clinical diversity, such variability is to be expected. What is significant is the identification that BQ has a measurable effect on oral pre-cancerous and cancerous lesions.

## Discussion

The IARC review concluded that areca nut is carcinogenic in humans and that it is linked to cancers of the oral cavity, pharynx, oesophagus, liver and biliary tracts, and the uterus (18). The global estimate of BQ chewing ranges between 10% and 20% with chewers concentrated in the South Asian and Pacific countries (19).

### Betel quid constituents

A trend that was consistent among some studies was the identification of several varieties of BQ use among different regions and the association between constituent variation and risk of OPMDs/MDs. Paulino et al. identified two types of BQ chewers in Guam and Saipan: the first type of chewers preferred the ripe, red or white areca nut and rarely if at all chewed immature fruits. This type also preferred to swallow the by-products and smoke cigarettes. The second type preferred the green, unripe areca nut and often combined it with betel leaf, slaked lime, and smokeless tobacco, and they tended to spit out the by-products. Interestingly, Paulino et al. found that more chewers of the second type had an OPMD compared with those of the first type (19.4% vs. 3.8%; *p* ≤ 0.01) (4). Similarly, Sari et al. found that West Papua residents had a higher OPMD prevalence compared with individuals from West Java and Jakarta. A later study by Sari et al. on the chemical profile of BQ types from different Indonesian regions confirmed that West Papua BQ, which consists of dried areca nut, slaked lime, husk, and betel inflorescence stem, had the highest arecoline and total phenolic content (14, 20). The four major BQ alkaloids known to cause mutagenic effects *in vitro* and *in vivo* are arecoline, arecaidine, guavacine, and guvacoline. A number of polyphenols also contribute to carcinogenesis (21). The metabolomic influence on BQ-related mutagenesis is suggested in many studies. For example, Loyha et al. investigated BQ constituents and their associated risk of MD, finding that red slaked lime contributed to the strongest risk of oral cancer compared with other BQ components (OR 10.67, 95% CI: 2.27–50.08) (11). The molecular constituents of BQ and thus the risk of OPMDs and MDs vary geographically because of the different ingredients and preparations used. Understanding this is a driving factor in determining disease-inducing capacity by the method of preparation (21). Paulino et al. (22) investigated BQ constituents in relation to demographics in Guam and Saipan. Dividing the results into a group of adult chewers (18–75 years old) and youth (9–17 years old), it was found that the top three chewing preferences for adults were areca nut with betel leaf, slaked lime, and tobacco; areca nut with slaked lime and tobacco; and areca nut with slaked lime and betel lead. On the other hand, youths mainly preferred only areca nut, although some chewed areca nut with slaked lime and tobacco and some also combined betel leaf to the mixture (22). This study was in line with that of Narayanan et al., who surveyed 300 participants over the age of 18 and found that 56.3% added betel leaf, 89.3% added slaked lime, and 84.7% of participants added tobacco to their BQ preparations (23).

Interestingly, the ABC consortium study noted that there is an emerging and high proportion of new BQ users from Hunan province in Mainland China. Chewers uniquely used the dried areca husk rather than the nut used by other countries (24). The result of a metabolomic profile test of husk found in Indonesia reveals that it contains the widest range of polyphenols compared with other constituents (20). Hunan chewers generally did not add tobacco to the mixture, but instead marinated the dried husk with slaked lime, sweeteners, cassia oil, and bittern (24). The husk is generally coarse and abrasive, leading to mucosal trauma. As a result, alkaloids and polyphenols diffuse better into the submucosal tissues, evoking an inflammatory response. To combat this, the body undergoes hyperplastic changes, which may accelerate or exacerbate the effects of OSF (25).

In Australia, a Burmese (Myanmar) community in Wollongong describes the use of areca nut, lime paste, and smokeless tobacco in their mixture, one that is identical to Myanmar's common BQ combination (26). Australian Indians also tend to chew BQ in the same way as in their home country, combining crushes or whole areca nut with slaked lime, tobacco, and betel leaf. In addition, commercially produced paan masala is available (5).

The freshness of BQ constituents and the addition of chemical preservatives varies both intra- and inter-regionally depending on the source. These additives, especially as BQ becomes mass-fabricated, may play a disease-inducing role. In Myanmar, increasing employee wages had resulted in ready-made, refrigerated, and packaged BQ, often wrapped in tobacco (27). This is also a common sight in countries such as Taiwan, where BQ is pre-packaged and sold in cardboard boxes to motorists by roadside vendors. Consumers are often blue-collar workers, relying on psychoactive stimulation for better work productivity. Those packages are often a combination of areca nut and slaked lime paste and may also include tobacco (28). Alternatively, local market vendors sell inexpensive BQ products, prepared to the taste of local users.

### Consumption

Consumption of BQ is generally through two methods: chewing and spitting, or chewing and swallowing the mixture. A study in Myanmar noted that BQ consumers generally spit the BQ after chewing; however, 1 in 10 would sometimes or always swallow it (2). The ABC study showed that chewers from Mainland China also swallowed the BQ, while the majority of chewers of SEA countries, including Taiwan, Malaysia, and Indonesia, tended to spit out the bolus (24).

Throughout Micronesia, chewing habits vary geographically because of the acculturation of migration patterns. In Yap, a Micronesian island, after the quid is chewed for a while, it is taken out and a few sprinkles of lime powder are added before chewing is resumed (29). This practice is alarming, because slaked lime expedites the hydrolysis of arecoline to arecaidine, drastically increasing fibroblast proliferation, increasing collagen formation, and amplifying the risk of oral cancer overall (21).

### Gender preponderance

Another common trend noticed in the studies was the gender preponderance. Lee et al. found that there were more male chewers than female chewers in Taiwan, Mainland China, while female chewers were significantly greater in Malaysia and Indonesia (3, 24). This was affirmed by the annual report of the 2019 Health Promotion Administration of Taiwan, which stated that around 970,000 Taiwanese adults are BQ chewers and men make up approximately 900,000 (30). Female predominance follows in Thailand, where the BQ chewing prevalence rate is 15.9%, of which 97.7% are women (31).

A study found that among 542 residents of a Myanmar township, 52% of the respondents chewed BQ, of which there was a much higher prevalence of male than female chewers (72% and 39%, respectively) (2). Among Saipan adolescents, BQ chewing was more prevalent in males than in females (32).

A Vietnamese retrospective study on cultural oral risk habits, including BQ chewing, found that from 2005 to 2006, 147 cases of OSCC were diagnosed, of which 100 were men and 47 were women. While more women with OSCC reported BQ chewing than men (40%), the most advanced stages of cancer were observed more in men than in women (33).

### Staging and types

Because of the fact that most screenings are performed in rural and remote townships with limited access to regular oral healthcare, MDs are usually identified at later stages, while a wide variety of OPMDs may be present due to a prolonged period of BQ chewing. In a Myanmar hospital, among 153 patients with head and neck cancer, 81.69% chewed BQ, often keeping it in their oral cavity even when asleep. While the buccal mucosa was the most common site (49%) in these patients due to the proximity to the BQ bolus, other sites of the oral cavity, as well as the larynx, were also affected. With regard to staging, 5.22% of patients were diagnosed in stage I, 33.33% in stage II, 46.4% in stage III, and 15.03% in stage IV. OSCC was the most common type of cancer (95.42%) (34). This trend of severe- and late-stage MD screening is concerning, yet it shows little improvement from a 1985 to 1988 hospital-based study in Myanmar where 71.4% of 70 oral cancer cases where found to indulge in BQ chewing. Of those cases, only 7.1% of cancers were screened in stage I, while an alarming 70% were screened in stage IV (1).

In the betel nut endemic–affected Commonwealth of the Northern Mariana Islands (CNMI), similar, alarming trends were noted in the year 2020. Of 55 patients with head and neck cancer, 53% were diagnosed with stage IV, with a 49.5% 5-year survival rate. Patients who chewed BQ were diagnosed at a significantly younger age that those who did not (47.2 and 55.4, respectively) (35). In 2022, a study by Duncan et al. characterising otolaryngology referrals among pacific islanders in the CNMI found that 52.2% of the adults referred reported a BQ chewing habit. Among 56 patients diagnosed with oral cancer, 94.5% were BQ chewers with a male predominance of 1.8:1 (36). Similarly, more than 7,000 oral cancer cases have been reported between 2012 and 2019, close to 90% of whom were BQ chewers. According to the Taiwanese cancer registry, the incidence of male oral cancer is 11.9 times than that for women due to the higher rates of chewing prevalent among men (30).

Mizukawa et al. screened BQ chewing endemic rural areas and in South Myanmar for suspicious lesions of oral and oropharyngeal cancers. Out of 105 screened subjects, lesions were detected in 39, two of which were oral carcinomas. The first case, a 58-year-old man with OSCC in the palate, had a history of chewing eight quids a day for 7 years as well as smoking and drinking. The second case, a 72-year-old woman, had OSCC in her lower gingiva. She reported chewing three to five betel quids daily for 10 years but denied smoking or drinking alcohol. Other subjects with lesions included seven with oral leukoplakia, with all of them chewing from 3 to 10 quids a day, but the majority did not smoke; four had lichen planus lesions, one an OSF lesion, and one had dysplasia (1). The consortium study takes a different approach, in that it details OLP, OSF, and OL presentation percentages per country. Interestingly, Indonesia had the highest overall prevalence of OPMDs among its abuse chewers: 15.7% had OLP, 8.8% had OSF, and 17.2% had OL. Taiwan had the second highest group of chewers with a disposition to OPMDs, with 5.4% of abuse chewers suffering OLP, 9.6 suffering OSF, and 3.8 suffering OL. Non-abuse Taiwanese chewers also had a higher disposition to OPMDs, bringing the overall OPMD percentage to 21.8. In Hunan, Mainland China, OSF was the most common OPMD affecting 5% and 5.6% of non-abuse and abuse chewers, respectively (37).

### Limitations

Limitations in this meta-analysis include comparing results with different statistical significance values and varying interpretations in adjusting the OR. Wherever possible, we attempted to exclude BQ mixtures with tobacco to limit confounding variables; however, this was not always possible as demonstrated. Most researchers of other studies did not transparently report their calculations or raw, crude OR, and therefore, it was not possible to statistically evaluate their findings. Ingredient comparison could not be performed comprehensively due to omitted information from our selected studies and could only be inferred based on the habits of the general population of interest. The different heterogeneities observed are also limitations of this study, possibly explained by the variability of demographics and conditions in different locations.

### GRADE evidence profile

The GRADE system was used to assess inconsistency, imprecision, indirectness, risk of bias, and other strengths and limitations of the studies that met the eligibility criteria for the meta-analysis (7, 8, 17). Three studies, namely, “Lin (2011),” “Lee (2012) Taiwan,” and “Lee (2012) China,” displayed very wide confidence intervals, leading to a high level of imprecision. Two studies, “Loyha (2012)” and “Amtha (2014),” contained very small sample sizes that were less than the recommended (>400) study size by the GRADE handbook, lending them high imprecision as well. Most studies accounted for confounding variables, with their adjustments reflected in their outcomes, and all studies contained positive gradients and large effect sizes because of widely reported ORs (38). This increased the quality of evidence to moderate, high, and very high for the articles included in the meta-analysis.

### Implications for further research in Australia

BQ chewing was previously thought to be concentrated in Asian and Pacific Islander countries where production is at its highest. It was believed that after production, the tobacco products are exported to neighbouring countries such as Australia and New Zealand to meet the demands of migrant residents. However, through our personal field observations, by searching Australian news articles and databases, and scouring social media, we found that our surmise was wrong.

**Figure 6:**
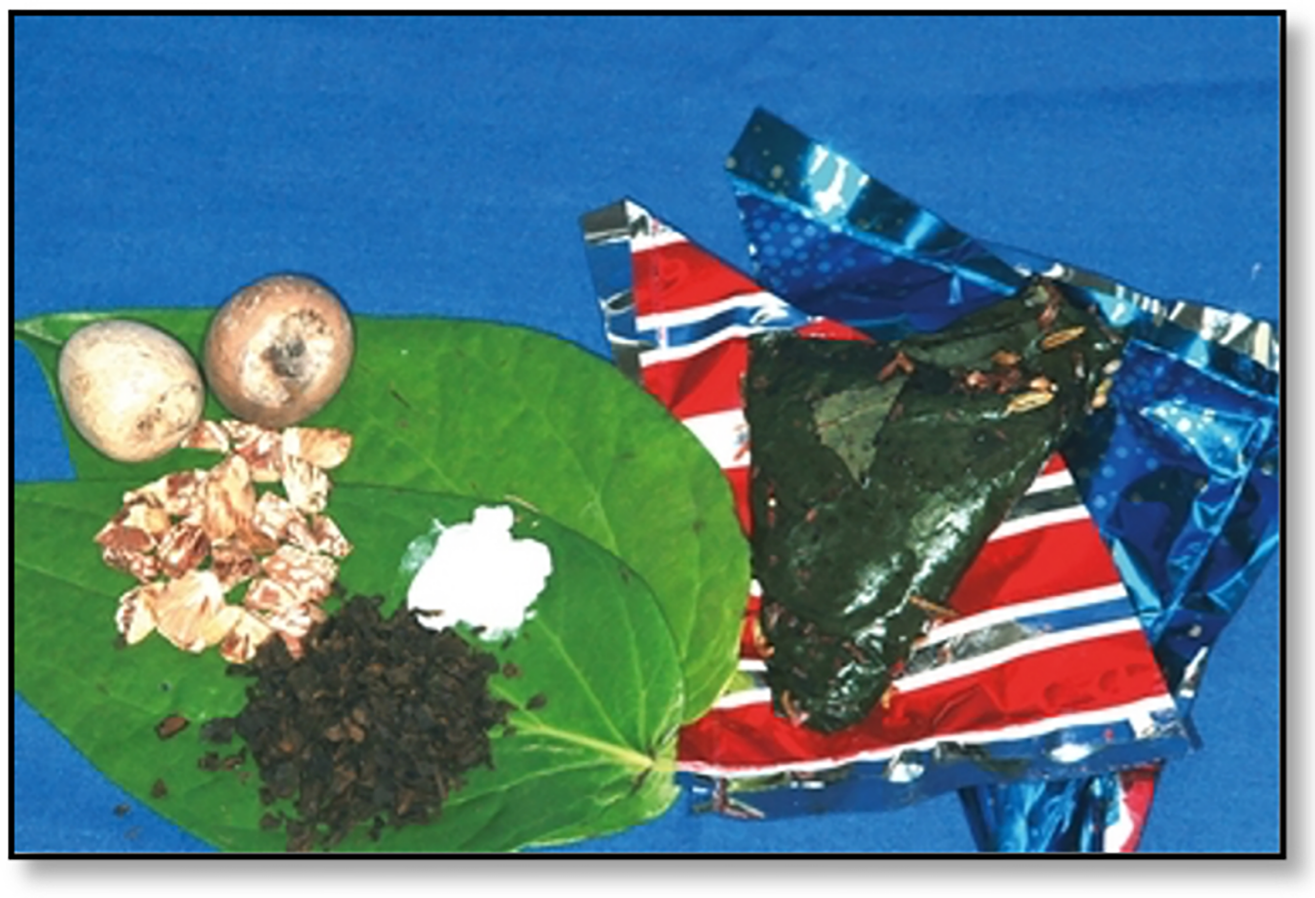
Areca nut, lime, betel leaf, tobacco, and paan masala from an Indian grocery store in Sydney (5).

**Figure 7:**
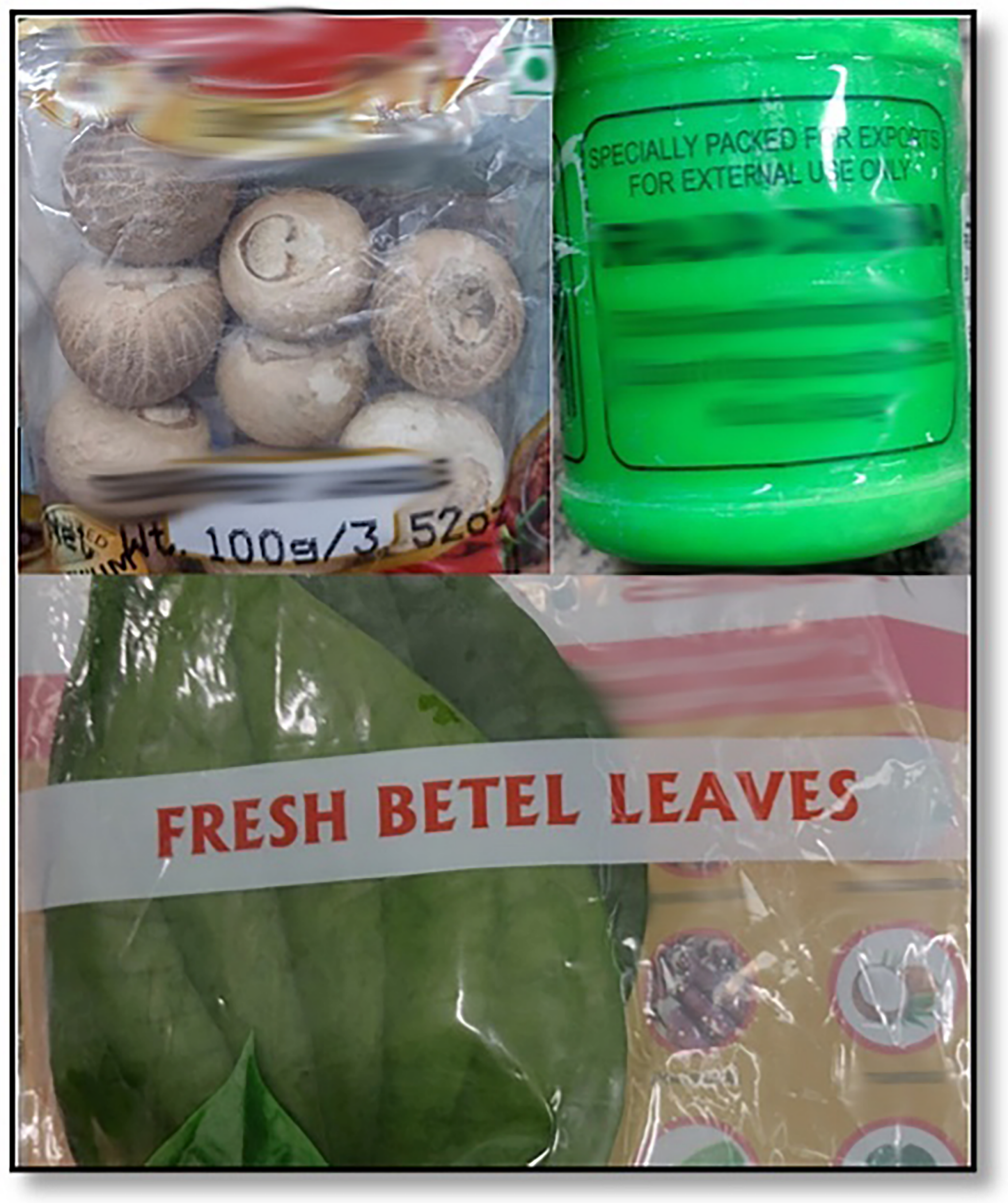
A betel quid package from an Indian grocery store in Melbourne containing dry, imported areca nut, betel leaf, and slaked lime.

**Figure 8:**
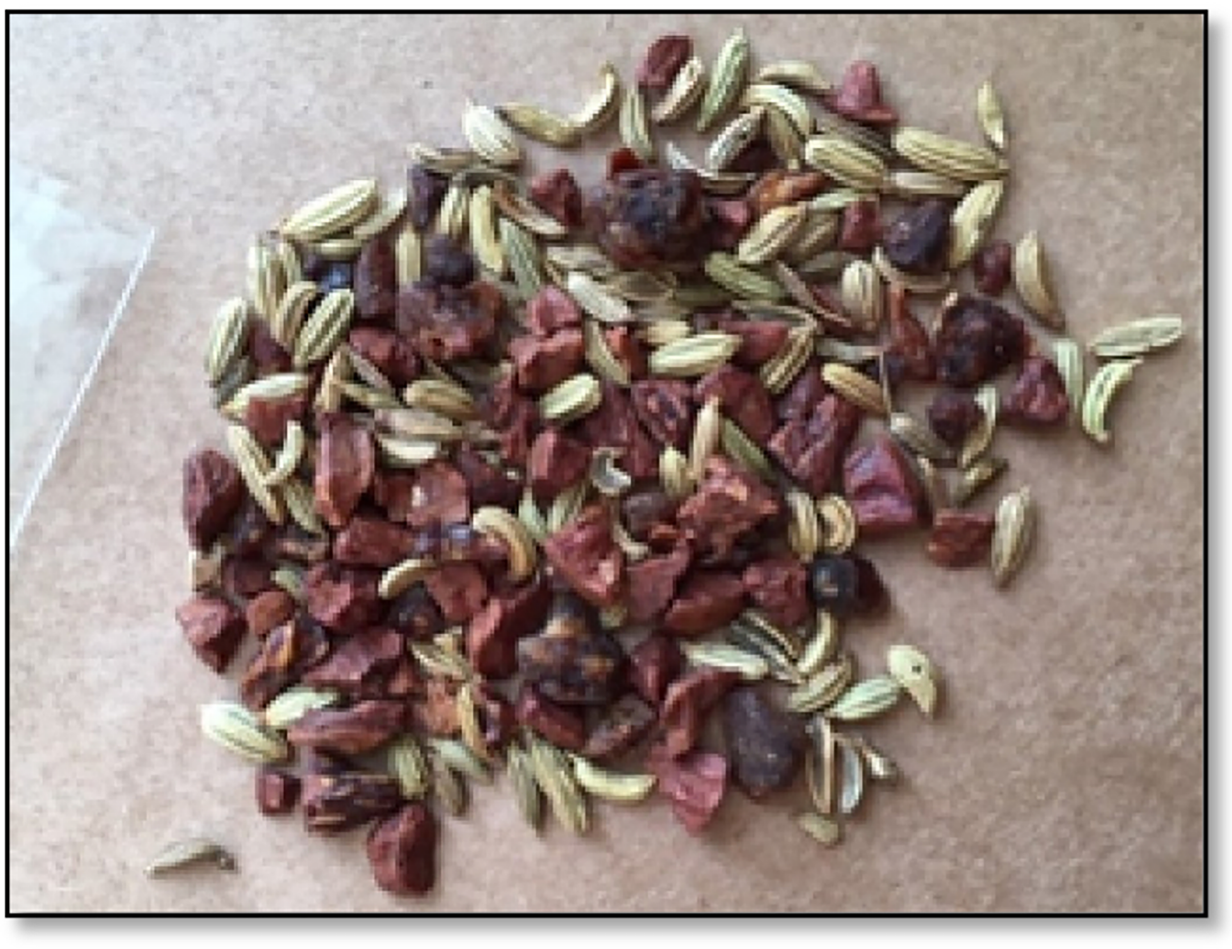
Betel quid sold in an Asian grocery store in Tasmania, Australia (39).

**Figure 9:**
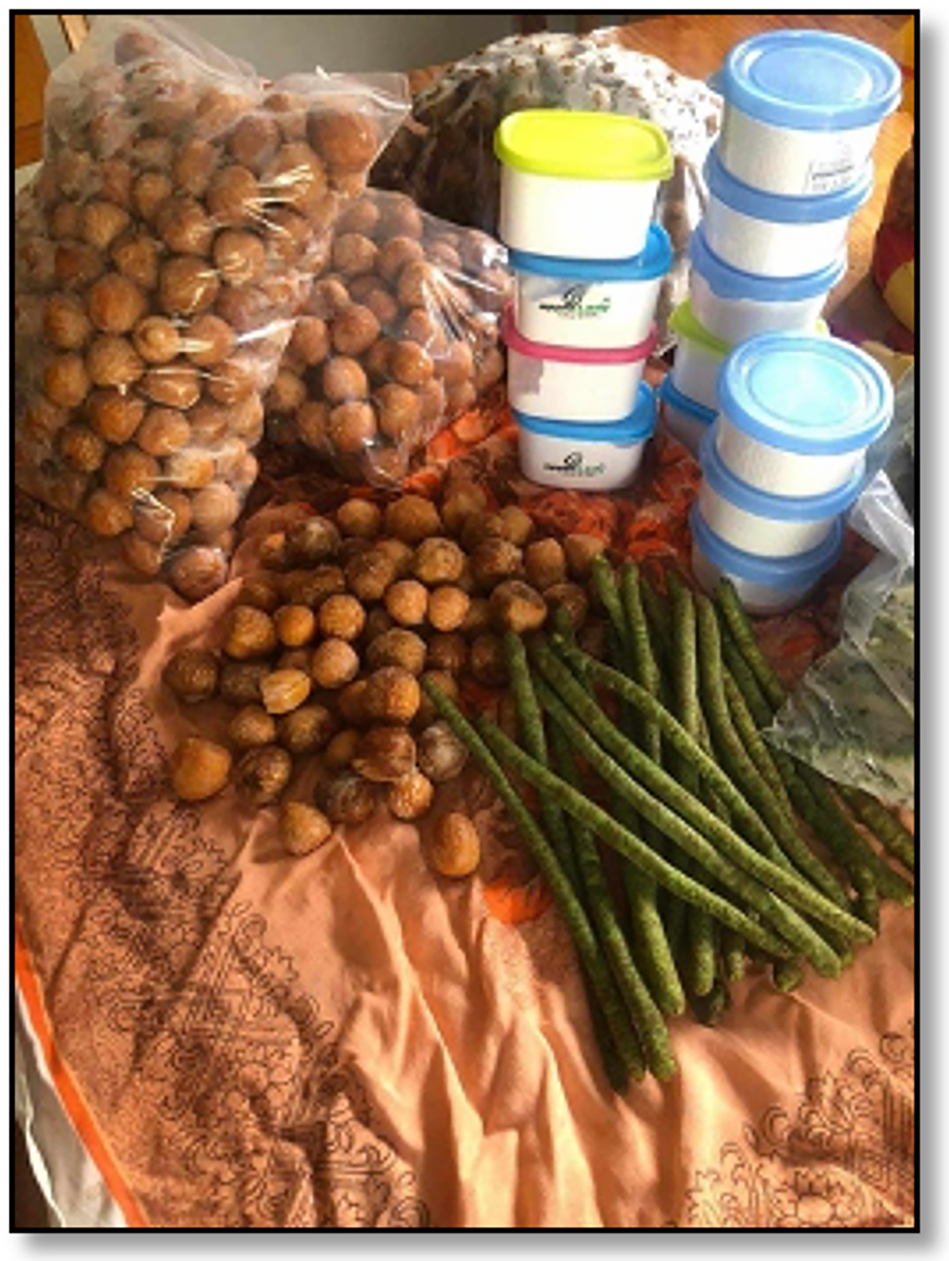
A betel quid package from Cairns, Australia, containing fresh, local areca nut, betel flower, and slaked lime (40). Betel quid seller © 2024 E Weeding.

BQ was found to be available to many populations and in outlets in Australia, most significantly, the Indian communities in the metropolitan areas of Sydney and Melbourne (Figures 6, 7), Asian groceries in Tasmania (Figure 8), and Papua New Guinea (PNG) communities, mostly concentrated in North Queensland and the northern parts of the Northern Territory(Figure 9).

Recent news articles on the chewing epidemic written in various media outlets, including the ABC, discuss the boom in black market sales as Australian laws bear down on local market vendors and Asian groceries (41, 42). As expected, no formal published articles on those “black markets” vendors were found, although through social media, we were able to locate them. We found that BQ ingredients such as the areca nut, the betel leaf or flower, and slaked lime were being produced locally on private properties in Northern Queensland, mainly in the Cairns region. These products were sold online and were to be possibly delivered to buyers from other states and territories in Australia, travelling as far as Melbourne. When screening the demographics of potential buyers on market posts, not all were of PNG descent, as it seemed that the influence and addiction of BQ was spreading to other local ethnicities, including the Caucasians. This observational finding may possibly turn out to be a paradox for dental professionals in Australia. The ethnicity characterisations of BQ chewing and OSF/OSCC will soon become blurred as demographically unpredicted patients begin to present with BQ-related oral manifestations.

Although no alkaloid and polyphenol concentration analysis has been formally performed on BQ mixtures available in Australia to predict their carcinogenicity, inferences may be made when comparing BQ mixtures with similar constituents in other countries. For example, it was found from our observations that Cairns-sourced BQ mixtures contained betel stem inflorescence, a mixture similar to the one chewed in West Papua, Indonesia. Interestingly, in the study by Sari et al., this region was also the most at risk of OPMDs (OR 15.18, 8.82–26.11) (43). This is supported by further research that betel inflorescence has a higher risk of developing OSF due to the higher concentration of polyphenols which act as pro-oxidants inside the highly alkaline environment created by slaked lime and produce carcinogenic factors in oral keratinocytes and oral fibroblasts (44).

Thus, interventional efforts become crucial before BQ chewing and its health consequences become widespread in Australia. Such efforts may help reduce the perceived burden of this habit on individuals, communities, and the economy. Understandably, BQ is a deeply ingrained cultural tradition; hence, educating chewers about less harmful BQ mixtures may be beneficial in order to reduce the risk of oral disease (44).

### Implications for practice and policy in Australia

Our research communicates an evidently strong association between BQ chewing and the risk of OPMDs and MDs, underlining the urgency of employing a harm-minimisation policy in common dental practice. This urgency is amplified when it is recognised that BQ ingredients are grown for people residing in regional/remote areas of Far North Queensland as well as being sold within immigrant communities—two very vulnerable groups facing health disparities (45). Currently, no guidelines or protocols for cessation of BQ are being used in Australia. Thus, we strongly recommend the introduction of BQ cessation protocols and guidelines in initial dental appointments. They may be initiated after evaluating a patient's consumption of BQ as part of the dental examination procedure. Upon identification of use, BQ cessation counselling and education should be offered and supervised across multiple sessions to aid in the prevention and harm minimisation of oral cancer. A structured, evidence-based module of BQ counselling needs to be created to aid the dental professional in facilitating positive change in the habits of their patients.

Currently, BQ is listed as a prohibited schedule 4 “poison” that is considered illegal to be consumed or sold under Australian legislation (46). However, the fact that the vast majority of BQ vendors were able to locate it easily in various Asian grocery stores in Melbourne, as well as over social media sites in other states of Australia, suggests ineffective regulations in controlling the production and selling of BQ. Therefore, we strongly recommend the implementation of more effective regulations to monitor BQ possession and distribution, which will help control the availability of this carcinogen to residents.

## Conclusion

This meta-analysis and scoping review clearly identify a strong association between BQ chewing, with or without tobacco, and the presence of OPMDs and MDs. This association still poses a great risk to the oral health and quality of life of chewers in the SEA and Pacific regions. While the habit may be decreasing in some regions, it has gained popularity among the local populace in other regions such as Cairns, Australia. This study delves deep into the rigid perceptions of BQ's continuous use among SEA and Pacific cultures, acknowledging the importance of understanding that constituents, methods of chewing, and gender may translate to the staging and types of OPMDs/MDs, suggesting a great benefit in harm-minimisation strategies to combat the BQ chewing endemic. There is a clear, overarching burden of OPMDs and MDs in the SEA and Pacific countries that will continue to result in high morbidity and mortality rates of oral cancer if no intervention to reduce disease risk is promptly and rigorously adopted.

## Data Availability

The original contributions presented in the study are included in the article/**Supplementary Material**, further inquiries can be directed to the corresponding author.
