# Supplementary material for: The association between betel quid use and oral potentially malignant and malignant disorders in Southeast Asian and Pacific regions: a systematic review and meta-analysis with GRADE evidence profile
Source: Front Oral Health. 2024 May 23;5:1397179. doi: 10.3389/froh.2024.1397179 (PMC11153687; doi:10.3389/froh.2024.1397179)
Supplement: Supplementary file 1 [file Table1.docx]

**Supplementary Materials**

**Table 1. Search Keywords**

| Categories | Entry Terms |
| --- | --- |
| Areca | Areca OR areca catechu OR areca catechus OR arecas OR betel nut OR betel nuts OR nut, betel OR nuts, betel OR catechus, areca. |
| Neoplasms | Benign neoplasm OR benign neoplasms OR cancer OR cancers OR malignancies OR malignancy OR malignant neoplasm OR malignant neoplasms OR neoplasia OR neoplasias OR neoplasm OR neoplasm, benign OR neoplasm, malignant OR neoplasms OR neoplasms, benign OR neoplasms, malignant OR tumour OR tumours |
| Precancerous Conditions | Condition, precancerous OR condition, preneoplastic OR conditions, precancerous OR conditions, preneoplastic OR precancerous condition OR precancerous conditions OR preneoplastic condition OR preneoplastic conditions |
| Australasia | Australia OR New Zealand OR Pacific Islands |
| Southeastern Asia | Borneo; Brunei; Cambodia; Indochina; Indonesia; Laos; Malaysia; the Mekong Valley; Myanmar (formerly Burma), the Philippines; Singapore; Thailand; Timor-Leste and Vietnam |
| Pacific Islands | Micronesia; Melanesia; and Polynesia (including New Zealand) |


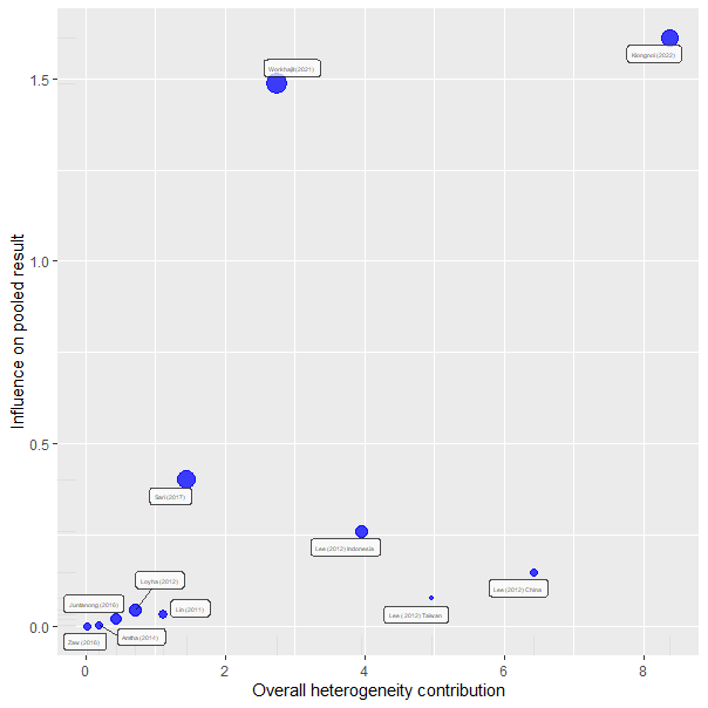


**Figure 1. Baujat plot graph**


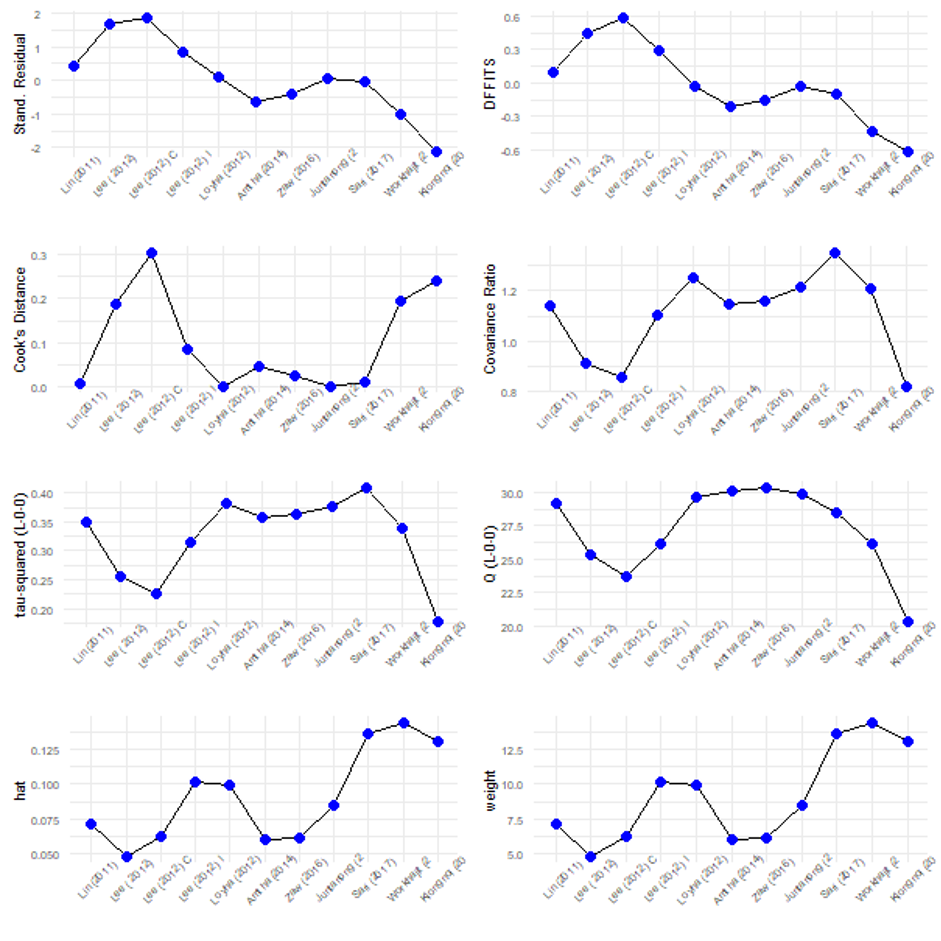


**Figure 2. Bimodal graph**


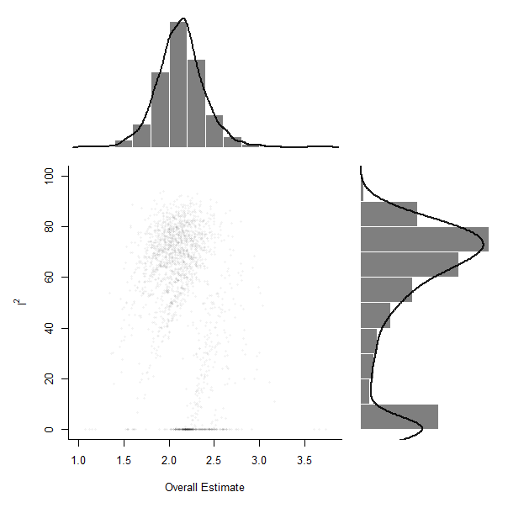


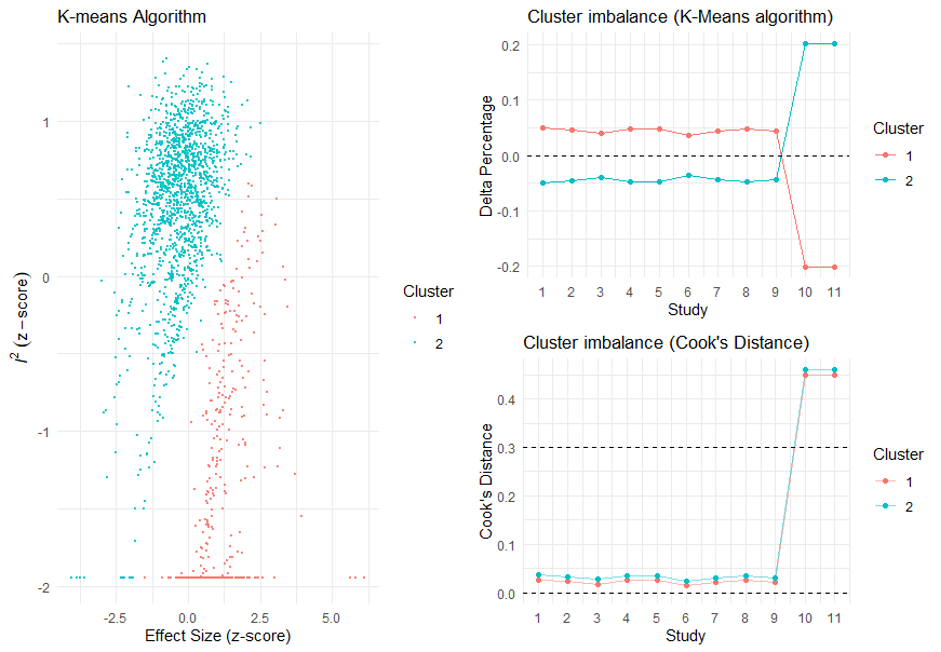


**Figure 3. K means Algorithm**


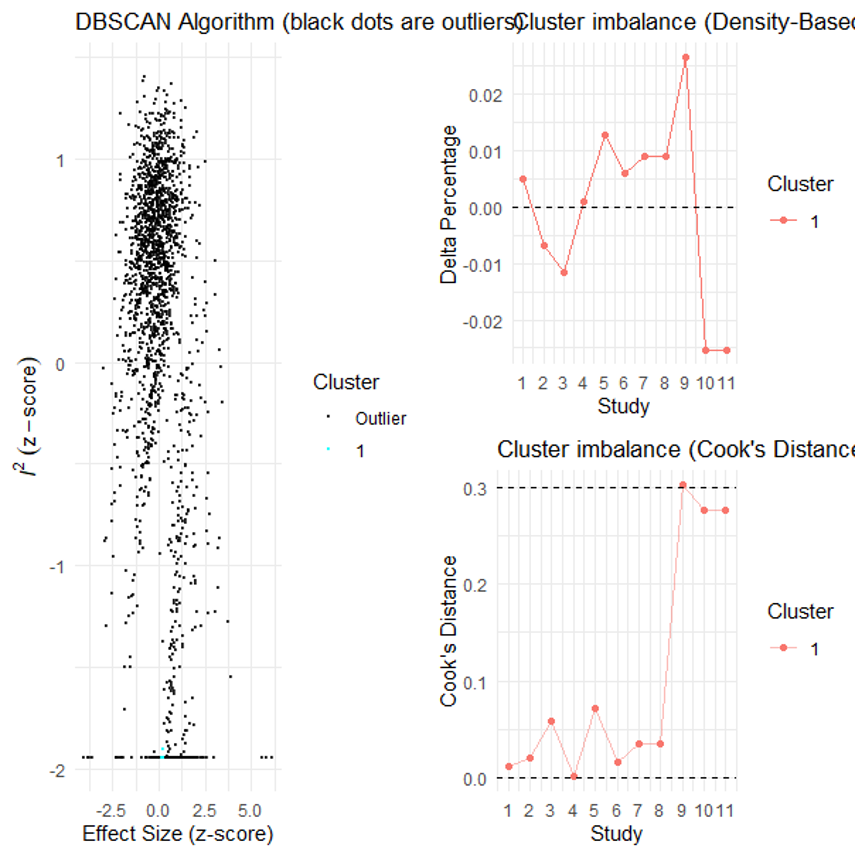


**Figure 4: DBSCAN Algorithm**


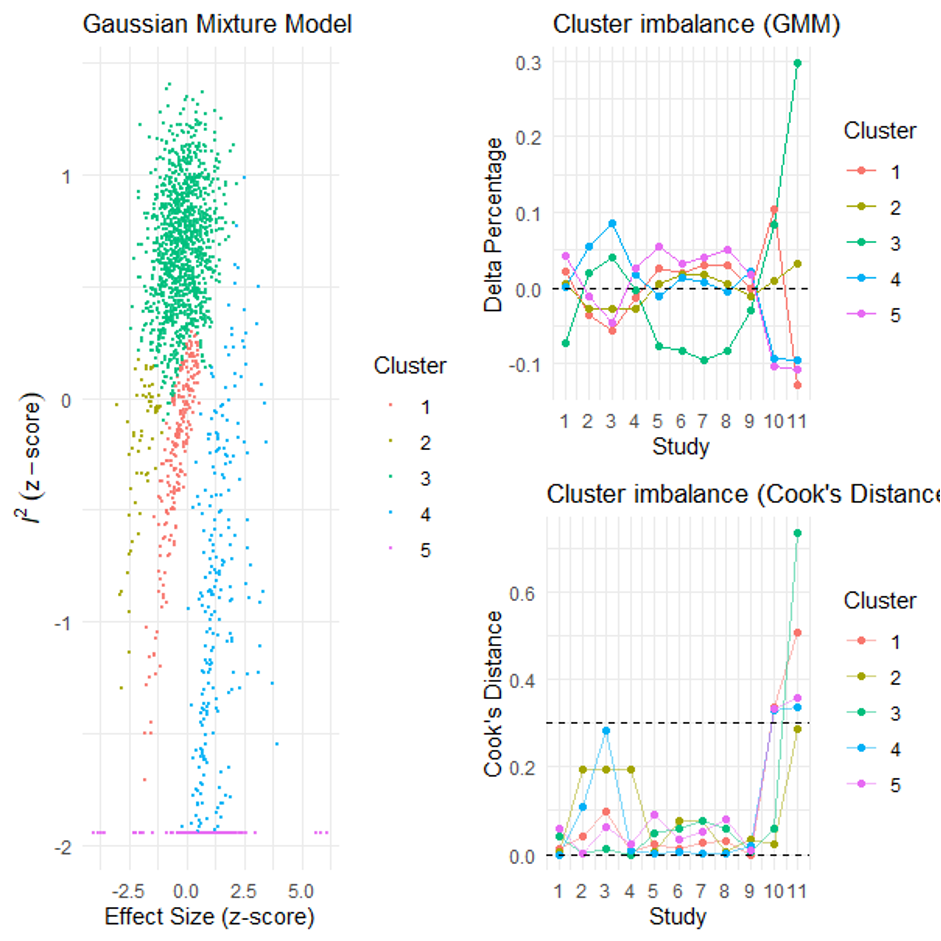


**Figure 5: Gaussian Mixture model**


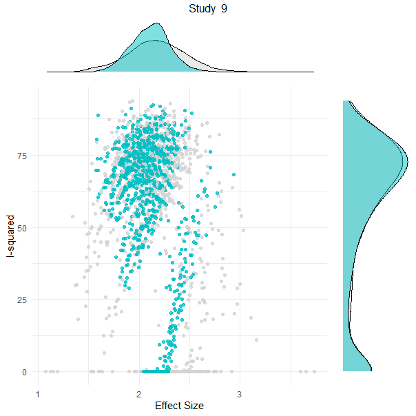

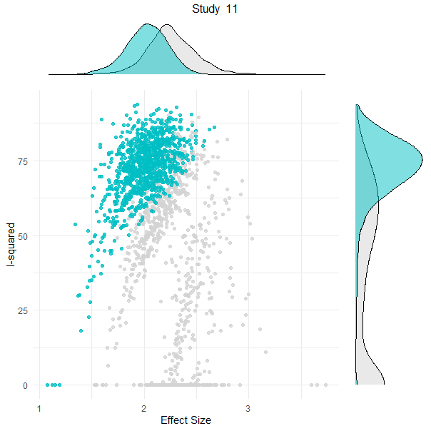

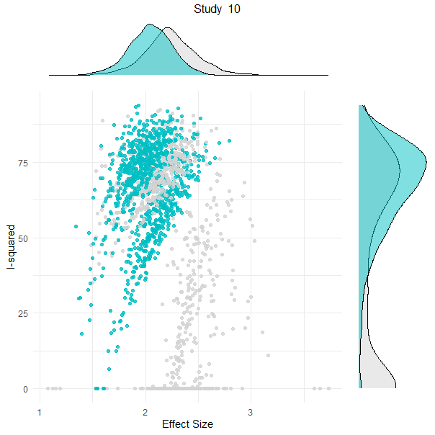


**Figure 6. Sensitivity test sorted by effect size.**


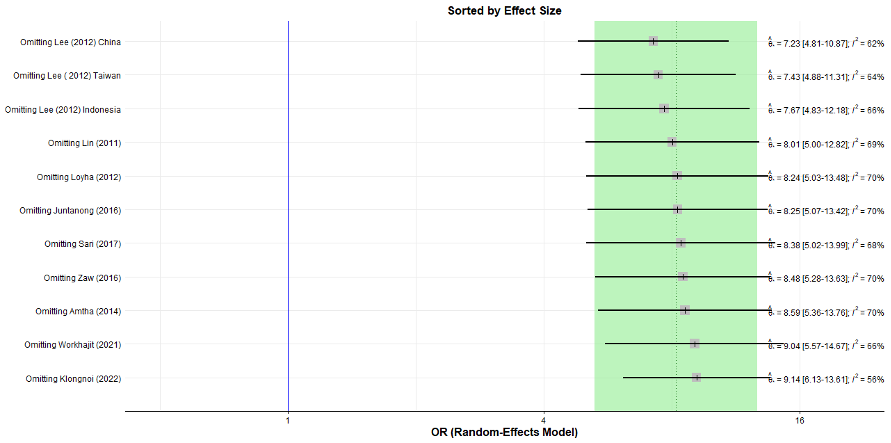


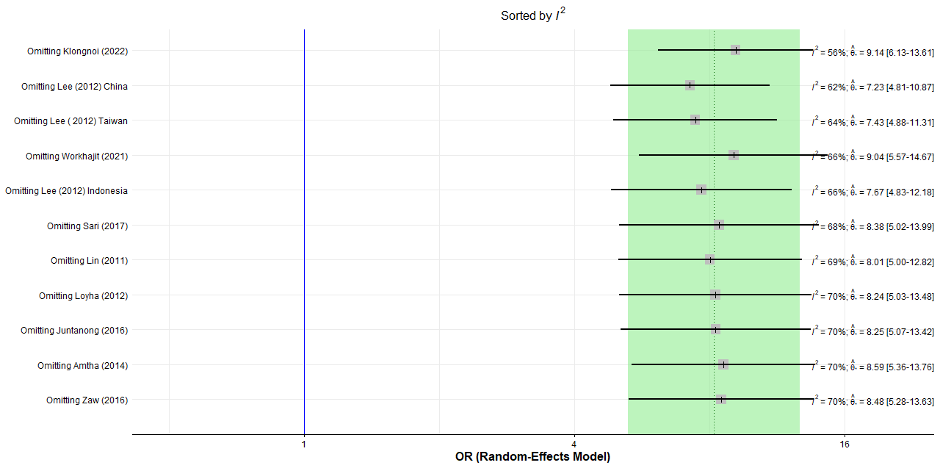


**Figure 7. Sensitivity test sorted by heterogeneity.**

**Eggers' test** of the intercept

=============================

intercept 95% CI t p

0.99 -0.66 - 2.64 1.18 0.2777

Eggers' test **does not indicate the presence of funnel plot asymmetry**.

**Figure 8: Eggers’ test of intercept**
